# Abstracts and Gleanings

**Published:** 1881-06-20

**Authors:** 


### ABSTRACTS AND GLEANINGS.

**Excision of a Portion of the Stomach and Duodenum.** In the Wiener Med. Wochenschrift, of February 5th, the following case is reported by Professor Billroth. The previous week a woman was brought to him having unmistakable symptoms of pyloric cancer. The patient, who was forty-three years old and mother of eight children still living, was attacked, apparently somewhat suddenly, with vomiting in October, 1880. All the symptoms of pyloric cancer soon developed themselves, and Dr. Billroth determined, with her consent, to operate, as she felt herself sinking under the increasing exhaustion and inability to retain food. The tumor lay on the upper side of the stomach and somewhat to the right; it seemed to be about as large as a moderate sized apple. A transverse incision, about eight centimetres (three inches and one-fifth) in length, was made over it through the wall of the abdomen. The tumor was difficult to disengage, on account of its size; it presented itself as a partly knotty, partly infiltrated cancer, covering the pylorus and rather more than a third of the under part of the stomach. Dr. Billroth loosened the adhesions to the omentum and the transverse colon, separated carefully the great and lesser omentum, and tied all the blood-vessels before cutting them through; the loss of blood was very slight. He then made an incision through the stomach one centimetre beyond the infiltrated part, at first in a backward direction only, and afterwards through the duodenum. Six sutures were then passed through the lips of the wound, the threads being left untied and only used to keep the lips of the wound in position. He then made a further oblique incision into the stomach from within and above in an outward and downward direction, keeping always one centimetre from the infiltrated part of the wall of the stomach, and then closed the oblique wound, from below upwards, until an aperture was left just of a sufficient size to fix the opening of the duodenum. The separation of the tumor from the duodenum was completed by means of an incision parallel to that in the stomach, and always at a distance of a centimetre from the infiltrated part. The duodenum was then introduced into the opening of the stomach which had been left. Altogether about fifty sutures were made with Czerny's carbolized silk. The wound was washed with dilute carbolic acid, and a few additional sutures inserted at weak points, the whole replaced in the abdominal cavity, and the abdominal wound closed and bandaged. The operation lasted an hour and a half. No weakness, vomiting or pain followed the operation. During the succeeding twenty-four hours the patient took only ice by the mouth and nutritive injections of wine; on the following day a tablespoonful of sour milk every half hour. The patient, a very intelligent woman, felt very well, and slept most of the night by help of a small injection of morphia. The piece excised was fourteen centimetres (about five and a half inches) in length along the greater curvature of the stomach. Only a quill could, with difficulty, be passed through the pylorus. The shape of the stomach is not much altered by the operation, but somewhat reduced in size. Sir H. Thompson, to whom we are indebted for the above, has received a note from Dr. Billroth, dated February 5th, the seventh day after the operation, in which he writes: "The sutures have been removed; the wound is healing without any reaction; the general condition of the patient is good; she takes broth and egg, coffee, tea and cocoa."—*Brit. Med. Jour.*

**Aphasia.**—Ferrier defines aphasia as follows: "The subject of aphasia is deprived of the faculty of articulate speech, and also very generally of the faculty of expressing his thoughts in writing, while he continues intelligently to comprehend the meaning of words spoken to him, or it may be to appreciate the meaning of written language. An aphasic individual knows perfectly well, as exhibited by his gestures, if a thing is called by its right name or not, but he cannot utter the word himself or write it when it is suggested to him. In his attempts, only an automatic or interjectional expression, or some unintelligible jargon escapes his lips, or unmeaning scrawls are set down on paper as writing. This affection is usually, at first at least, associated with a greater or less degree of right hemiplegia, but the motor affection of the right side, chiefly of the right arm, is often slight and transient, or may be wanting from the first, the only indication of motor paralysis being a paretic or weak condition of the oral muscles of the right side. The inability to speak is not due to paralysis of the muscles of articulation, for these are set in motion and employed for purposes of mastication and deglutition by the aphasic individual. The cause of the affection was shown by Broca (and his observations have been confirmed by thousands of cases) to be associated with disease in the region of the posterior extremity of the third left frontal convolution, where it abuts on the fissure of Sylvius and overlaps the island of Reil." "One of the most common causes of the affection is softening of this region, consequent on sudden stoppage of the circulation by embolic plugging of the arterial channels which convey its blood supply, by which the functional activity of the part is temporarily or permanently suspended." "The rapid recovery which so frequently occurs in cases of aphasia, especially of the kind due to embolic plugging of the nutrient arteries of the left centres, is not so much to be regarded as an indication of the education of the right centres, but rather of the re-establishment of the circulation and nutrition in parts only temporarily rendered functionless."—*Boston Medical Journal.*

**Erysipelas—Case in Practice.**—Mr. N., aged about 38; nervous, sanguine temperament. Was in good health on the evening of April 3, 1880; on the morning of the 4th, felt slight pain in the head and right side of the neck, about one inch below and back of the ear. During the day the pain increased. At five o'clock P. M., erysipelatous inflammation spread over the entire right side of the face and head. Pulse, 100. Gave aconite, 6, ten drops in two-thirds of a tumbler of water, a teaspoonful every fifteen minutes for one hour, then discontinue two hours, then repeat the remedy, to be continued in the same manner until ordered otherwise. At nine o'clock A. M., the 5th, both eyes were closed, and the inflamed surface presented a fiery red appearance; pulse, 120; tongue dry and red. Aconite continued. A topical application was applied to the inflamed surface, consisting of the proportion of one spoonful of alcohol to three of water, and to that was added two drops of the 6 of aconite to the spoonful of the lotion, and applied by wetting four thicknesses of cotton cloth and enveloping the inflamed surface, and renewed frequently. At nine P. M., the swelling and redness have continued to extend, assuming a deep, dark-red color. Same treatment continued. At seven A. M. of the 7th, pulse 125; pain in the head much worse among the parotid and salivary glands. During the day pulse run up to 130. Treatment continued. At six A. M. of the 8th, swelling at a stand-still; pain in the head not so severe. At seven P. M., pulse 100; swelling decreasing; all the symptoms more favorable, as far as I could see. At two A. M. of the 9th, the patient had a sinking chill; had great distress from severe cramp-like, burning pain in the region of the cardiac orifice of the stomach. The extremities cold, the pulse very feeble and fluttering; purple appearance of the hands and feet; cold perspiration over the body; frequent attacks of nausea. Gave arsenicum, 30th, the one quarter of a grain every fifteen minutes. In thirty minutes was much better, and within the hour the chill had entirely disappeared.

The reaction brought the pain back with an increased heat and redness of the inflamed parts. At nine A. M., of the 9th, pulse 140, attended with a low, muttering delirium. Gave bell., 6, ten drops in two-thirds of a tumbler of water, a spoonful every fifteen minutes for an hour; then discontinued an hour; then gave an hour, and discontinued two hours, and so continued the time to give; and also reapplying the lotion as at first. At nine P. M., delirium gone, pulse 120, swelling decreasing. At seven A. M. of the 10th, eyes partially opened, pain in the head gone, some appetite, little perspiration, tongue moist and of natural appearance. Same treatment continued. From this date the patient makes a rapid recovery. Dismissed on the 16th.—*Medical Call.*

[The above case we extract from a Homeopathic Journal, that our readers may see an example of how a homeopath treats a case of erysipelas.—ED. RECORD. W.J

**Viburnum Prunifolium.**—The black-haw bush, or small tree, everybody knows; but medicinally, very few know that the profession have in it a real remedy in threatened abortion, or flooding after it. I was called to see a lady in her seventh month of pregnancy, with violent pains coming on every five minutes, and which had been increasing for several hours. I gave her at once one drachm of the fluid extract, with thirty grains of hydrate of chloral. In an hour the pains moderated some little, and I repeated the viburnum, with twenty grains of chloral. Two hours after I gave the same, and the pains subsided. The patient slept several hours. In six or seven hours the pains returned again, and I again gave one drachm of viburnum and twenty grains of chloral; I gave three doses, subduing the pains. Being called away to a labor case, I was absent *twelve* hours, and being sent for hurriedly, I found my patient, as before, with more violent pains, and the os uteri opened three-fourths of an inch. I

repeated the same doses four times, and the pains subsided. This condition continued about eight days, but required less chloral each day. Every three or four hours I gave milk freely, keeping the bowels open by enemata. The patient bore the medicine well, and made a good recovery, and two months afterwards went through her labor satisfactorily.

I had often tried hydrate of chloral and other medicines, vainly, to check abortion or miscarriage, after the womb commenced opening.

Two months afterward I was called to see a similar case in threatened abortion, with flooding. I gave the viburnum alone, as I desired the patient to be awake in order to report hemorrhage. In two hours the pains and hemorrhage both ceased, with good recovery.

I was called last year to another patient—flooding dreadfully—and the contents of the womb were partially removed. I gave viburnum and ergot, and used hot-water injections with the bag syringe (otherwise called fountain syringe)—a great improvement on the rubber bulb. The flooding was violent, and required continuous use of the syringe and medicine for two days before the hemorrhage ceased. The abortion was complete.

A short time since I was called again to see the same lady, in her seventh month of pregnancy, with violent pains every seven minutes, but no flooding. I gave viburnum and chloral as before, but the stomach rejected three doses in succession. I then gave four drachms of viburnum and eighty grains of chloral by enema. In one hour the pains moderated somewhat. I gave half the quantity for the second dose, and the pains gradually stopped without further trouble. I used the fluid extract prepared by Parke, Davis & Co., Detroit.

If this preparation is not accessible, I would use the decoction of the fresh bark. The profession can rely on this remedy, and doubtless many lives will be saved by its prompt use.—*Dr. Cullen in Med. Herald.*

**Fractures of the Forearm.**—There is but one mode of dressing necessary for all fractures of the forearm, whether these be of one bone or of both, and whatever be their situation.

*Method of Dressing.*—The pieces for dressing a fractured forearm consist, first, of cotton batting; second, of light wooden splints; third, of bandages. The splints should extend from the elbow to the tips of the fingers; they should be a trifle wider than the wrist, to prevent lateral pressure upon the bones, and the obliteration of the interosseous space; they should not be much wider, else lateral displacement may occur. For convenience they may be shaped to the arm and hand. It will always be found more convenient to envelope the arm with the cotton, instead of padding the splints with the same; and where splints are padded with the cotton, it is always better to fasten this material by a few turns of ordinary sewing thread. The method of padding splints by securing the cotton with bandages, interferes greatly with their plasticity and comfort.

The bones, having been put in apposition by gentle extension, and the splints secured to the palmar and dorsal aspect of the arm by proper bandaging from tips of fingers to elbow; the arm is to be placed in a sling with thumb pointing upwards, in which position the bones are half way between supination and pronation, and the interosseous space is well preserved. The dressing, when fitted for fracture of the forearm is not to be removed, if comfort declares that it is properly doing its work, for a week or ten days, when the splints are to be shortened, so that they shall not reach beyond the roots of the fingers—and these are to be exercised frequently to prevent stiffness. The interosseous pad, formerly considered necessary to preserve the interosseous space, is very nearly obsolete, and should be entirely so.

The pistol-splint does nothing toward preserving the interosseous space.

The complicated dressings for Colles' fracture of the radius are not called for, and such dressings as include a compress to correct deformity are to be condemned as unsurgical, not only at the wrist, but anywhere.

In Colles' fracture, after union has taken place, there frequently remains some of its characteristic deformity. In the young, this generally disappears under the play of the muscles, or sometimes in bone recently united, it may be remedied by actual compression.

A common result in Colles' fracture, and in fractures near the wrist in adults, and especially in the aged, is a severe and persistent neuralgia. It is best treated by the hot-water douche.

In fractures of both bones of the arm, and frequently after fracture of one bone, after union, there is a bowing of the forearm always toward the ulnar side. Sometimes this is chiefly apparent, often real. It will frequently disappear, even when excessive—under the play of the muscles, especially in the young.—*Dr. Cowling at Kentucky Medical Association.*

**Hypodermic Injection of Atropiæ Sulphur—A Specific for Sciatica.**—Dr. Smythe, in Wolsh's Retrospect, claims this to be a specific in sciatica.

This remedy must be used in full doses. If less than the 1-24 to 1-12 of a grain be used, the success will only be partial, and after the patient has suffered from the drug once without relief, it may take some persuasion to make him undergo it again. I tried the effect of a small dose, 1-40 of a grain, in one case. The relief was only partial, and it was several days before the consent of the patient was obtained for a repetition of the remedy. The 1-16 of a grain produced a permanent cure, since which time I have never used less quantity.

The remedy must not be used when the system is under the influence of opium or any of its preparations, owing to the antagonism known to exist between these drugs.

If any dangerous symptoms should appear, they are readily counteracted by any of the preparations of opium. In none of my cases did I deem it necessary to use the antidote.

I tried this remedy in two cases of gonorrhœal sciatica, without any permanent relief. The medicine was administered three times in one case and twice in the other, producing its full constitutional effect each time, hence I am forced to the conclusion that this rare form of disease does not yield to the remedy any more readily than gonorrhœal rheumatism will yield to salicylic acid.

Allusion is made to one distressing case in which one-twelth of a grain of sulph. atropiæ was injected beneath the skin immediately over the tender spot in the nerve, at 3 o'clock P. M., or about an hour before the exasceration. Patient had all the constitutional symptoms of belladonna poisoning developed in a moderate degree: dilatation of the pupils, disturbance of the vision, and dryness of the throat; oppression of the chest, iron bands around the forehead, delirium and so on. These symptoms subsided in about nine hours and left patient comfortable and entirely free from pain which never returned.

I saw the patient at noon on the following day and found him, to my surprise, walking about the ward without pain or inconvenience, the contracted muscles having fully relaxed, and good motion restored to knee-joint in less than twenty-four hours after the hypodermic injection was administered.

**How to Preserve the Teeth.**—(1.) The teeth should be cleaned at least once a day, the best time being night—the last thing. For this purpose use a soft brush, on which take a little soap, and then some prepared chalk, brushing up and down and across. There is rarely any objection to the friction causing the gum to bleed slightly.

(2.) Avoid all rough usage of the teeth, such as cracking nuts, biting thread, etc., as by so doing even good, sound teeth may be injured.

(3.) When decay is first observed, advice should at once be sought. It is the stopping in a small hole that is of the greatest service, though not unfrequently a large filling preserves the tooth for years.

(4.) It is of the greatest importance that children from four years and upwards should have their teeth frequently examined by the dental surgeon, to see that the first set, particularly the back teeth, are not decaying too early; and to have the opportunity of timely treatment for the regulation and preservation of the second set.

(5.) Children should be taught to *rinse* the mouth night and morning, and to begin the use of the tooth-brush early (likewise the tooth-pick).

(6.) With regard to the food of children, to those who are old enough, whole meal bread, porridge and milk should be given. This is much more wholesome and substantial food than white bread.

(7.) If the above instructions were carried out, comparatively few teeth would have to be extracted.

**Sand Bag for the Sick-Room.**—One of the most convenient articles to be used in a sick-room is a sand bag. Get some clean, fine sand, dry it thoroughly in a kettle on the stove; make a bag about eight inches square of flannel, fill it with the dry sand, sew the opening carefully together, and cover the bag with cotton or linen cloth. This will prevent the sand from sifting out, and will also enable you to heat the bag quickly by placing it in the oven, or even on top of the stove. After once using this you will never again attempt to warm the feet or hands of a sick person with a bottle of hot water or a brick. The sand holds the heat a long time, and the bag can be tucked up to the back without hurting the invalid. It is a good plan to make two or three of the bags and keep them ready for use.—*Chicago Medical Times.*

**Morphia and Chloroform Combined, for Anæsthesia.**—Dr. Alex. Crombie, of Bengal, urges in *The Practitioner*, December, 1880, the administration of chloroform by inhalation, and, *as soon as slight insensibility is produced*, the hypodermic injection of about one-sixth of a grain of muriate of morphia. His experience has been that, by this method, prolonged anæsthesia is produced with an extremely small quantity of chloroform. Vomiting and asphyxia he thinks are much less likely to occur than when chloroform alone is used, and the fact that insensibility is maintained for some time without the inhalation of chloroform makes this plan specially valuable in operations about the face.

He recommends, also, a method for keeping open the passage to the larynx, which, though not new, is not so well known as it should be, for it is of great practical value. This consists in thrusting forward the lower jaw, by pressure against the ascending rami, until the lower teeth overlap the upper. By so doing, the tongue and hyoid bone are dragged forward more effectually than by the plan of pulling the tongue forward.—*Specialist.*

**Aromatic Tetrachloride of Carbon.**—Tetrachloride of carbon was brought into notice as an anesthetic and sedative by Dr. Protheroe Smith, in a series of papers which appeared in the Lancet of May and June, 1867. It is a colorless, exceedingly volatile liquid, having a delicate odor not unlike that of quince. From Dr. Protheroe Smith's papers it appears that, when inhaled, this body is very effective in removing the pains of labor and of dysmenorrhea, and that it has been extensively and successfully employed for headache, toothache, neuralgia, lumbago and rheumatic pains, applied externally on American leather cloth. Dr. Protheroe Smith has found it very efficacious in the mitigation and cure of hay-fever. He usually prescribes a very purified and perfumed compound manufactured by Corbyn & Co., which he calls *Aromatic Tetrachloride of Carbon*, and for which there is considerable demand. "This new sedative, to which Dr. Protheroe Smith called attention in the Lancet about ten years ago, is prepared in a very agreeable form by Messrs. Corbyn. It has an odor like that of quince, and is said to have been useful in removing the pains of labor and dysmenorrhea, in hay-fever, and in neuralgia."—*Lancet.*

**Hypodermic Injections of Coffee.**—Dr. M. A. Pallen, of New York, has had occasion to inject a solution of coffee subcutaneously in two cases. These injections were given to control the vomiting that had been excited by previous doses of morphia. The effect is tonic and stimulating, and promptly sedative to stomach irritability. In one case an over-dose of morphia had been taken, which was followed by the usual narcotic effects, and thirty minims of strong fluid extract of Java coffee introduced into the abdominal parieties stimulated effectively. After a short interval another subcutaneous injection was made a little above the epigastrium, and finally another in the right arm over the deltoid. Abscess followed the use of the coffee when used cold, but when warmed slightly to the temperature of the blood it never caused abscess in Dr. Pallen's practice.—*Journal of Materia Medica.*

**Usual Situation of Diphtheritic Membrane.**—Dr. Chisolm reported two cases of diphtheritic deposit confined exclusively to the lower eye-lids. The glands at the angle of the jaw were enlarged. There was no febrile excitement. Iodoform locally applied caused rapid disappearance of the membrane.

Dr. Arnold mentioned the case of a child who, three days after the operation of circumcision, was taken ill. A large diphtheritic patch formed on the wound. The cervical glands were enlarged, although nothing was to be seen on examining the throat. The glands in the groins were not enlarged. The child gradually sank and died. Diphtheria was present in the neighborhood at the same time.—*Virginia Medical Monthly.*

**Germany has 8,404 Doctors of Medicine,** of which 944 are at Berlin, under legal direction, with medical judges. It may have defects, but better to live with defects than not at all.—*La France Medicale.*

**Boracic Acid for Cholera.**—The London Lancet calls attention to the value of boracic acid in cholera, as exhibited in the cases treated by Surgeon Butler, of the Madras Medical service. It was, it appears, at the period when the properties of boracic acid were first made public, that Dr. Butler determined to try its effects in this direction. The pure acid not being procurable, the biborate of soda (borax) was at first employed, and with marked benefit, the percentage of recoveries being from 70 to 75 per cent. Subsequently he used the pure acid in ten grain doses every two hours, combined with borax or biborate of soda, under which treatment every case recovered. Dr. Butler further asserts that in no case were any signs of irritation or ill effects observed from the remedy, and in all of them the renal secretion was re-established with much greater facility than under any other method.—*Ibid.*

**Heroic Police.**—A few days ago, about 8 o'clock in the morning, the people on upper Broadway were startled by beholding a negro running down the street stark naked, and pursued by a large crowd of men and boys shouting "small-pox!" He was quickly captured, however, by two policemen, who threw a horse-blanket over him, and took him to a neighboring station-house, whence he was presently conveyed to the reception hospital, at the foot of East Sixteenth street. It seems that the man had gone to bed not feeling well the evening before, and that during the night confluent small-pox had developed itself. In the morning, in the violent delirium peculiar to the disease, he had sprung out of bed, torn off his night-shirt, and rushed into the street naked.—*Cin. Clinic.*

**Cure for Night-Sweats.**—A powder, composed of 3 parts salicylic acid and 87 parts of magnesium silicate, is used in the German army as a remedy for sweating of the feet. Recently a Belgian physician tried its efficiency in several cases of night-sweating by consumptives. The beneficial effect was immediate and permanent. The powder was rubbed over the whole body. During the application the mouth should be protected with a handkerchief.—*Scientific American.*

**The Wet-Sheet in Pneumonic Fever.**—Dr. Austin Flint, in a recent clinic (Gaillard's Medical Journal, March, 1881), reported three cases of pneumonic fever, treated by means of the wet-sheet. The plan of treatment was as follows:

The directions were to employ the wet-sheet whenever the axillary temperature exceeded 103° Fahr. The patient was wrapped in a sheet saturated with water at a temperature of about 80° Fahr., the bed being protected by an India-rubber covering. Sprinkling with water of about the same temperature was repeated every fifteen or twenty minutes. If the patient complained of chilliness, he was covered with a light woollen blanket, which was removed when the chilly sensation had disappeared. In none of the cases was the blanket used much of the time while the patient was wrapped in the wet-sheet. The patient remained in the sheet until the temperature in the mouth fell to 102° or lower, care being taken to watch the pulse and other symptoms. When the temperature was reduced, the wet-sheet was removed, and resumed if the temperature again exceeded 103° Fahr.

The first case entered the hospital on the third day after the attack. On the second day after his entrance the wet-sheet was employed thrice. He remained in the sheet the first time, two hours and forty-five minutes; the second time an hour and a half, and the third time an hour and ten minutes. On the second day the wet-sheet was employed once, and continued one hour. On the third day, the wet-sheet was not employed, the temperature not rising above 103. On the fourth day, the wet-sheet was employed once, and continued for an hour. There was complete defervescence on the fifth day, and no return of the fever afterward. Dating from the attack to the cessation of fever, the duration of the disease was seven days. The patient had no treatment prior to his admission into the hospital. The treatment in the hospital, in addition to the employment of the wet-sheet, consisted of carbonate of ammonia in moderate doses, whisky given very moderately, and a little morphia. The patient was up and dressed five days after the date of the defervescence. There were no sequels, and the patient was discharged well.

The second case entered hospital seven days after the date of the attack. She had no medical treatment prior to her entrance. The wet-sheet was employed on the second day after her admission, and continued for six hours. Complete defervescence took place on the third day. Recovery followed without any drawbacks. Both lobes of the left lung were involved in this case. The invasion of the second lobe, probably, was about the time of her admission into hospital.

The third case entered hospital three days after he was obliged to give up work. On the day of his entrance the wet sheet was employed and continued ten hours. The wet-sheet was employed on the second day after his admission, and continued for five hours. Defervescence took place on this day. The duration of the fever was five days, dating from the time he was obliged to give up work, and seven days from the occurrence of chills and pain in the chest.

Dr. Flint said the histories of these cases as bearing upon the treatment employed, were of considerable interest. They certainly show that in cases like those which were selected, the treatment is not hurtful. More than this, they render probable the inference that the disease was controlled and brought speedily to a favorable termination by the treatment. They also go to show that the disease is essentially a fever, and that treatment is to be directed to it as such, and not as a purely local pulmonary affection. It remains to be determined by further observations how often and to what extent this method of treatment has a curative efficacy. It is also an important object of clinical study to ascertain the circumstances which render the treatment applicable to cases of pneumonic fever, and, on the other hand, the circumstances which may contra-indicate its employment in this disease.—*News and Abstract.*

**Sodium Phosphate in Habitual Colic.**—Dr. R. N. Taylor writes in the Medical Herald:

"It has fallen to my lot to have been beset with a number of patients who would persist in having colic at the most inopportune moments imaginable; first one, and then the other, then still another one, these colicky friends of mine were constantly interfering with my professional engagements, and what was more important still, with my hours of rest. Without entering into a discussion of the pathology of these cases, I beg leave to present in brief the history of one of the worst I have ever seen, together with the treatment, which resulted in completely warding off the attacks in this case, as in all others in which it has been carried out:

"Mrs. H., aged seventy-two years, for fifteen years has been the subject of attacks of cramp colic, recurring at first every two or three months, but for six years past coming on every two weeks, sometimes every week; has taken nearly everything in the materia medica, both at the hands of regulars and quacks, without any benefit, the attacks continually recurring, and that without any regard to errors of diet, coming on at nearly regular intervals, no matter how careful or how particular she might be in regard to her diet.

"During the paroxysms of colic the pain is most attrocious, accompanied by cramping pain in the extremities, nausea, vomiting, etc., to such an extent as to demand my immediate presence, armed with the hypodermic syringe.

"This patient was put upon the use of phosphate of soda, grs. xxx., ter in die, before meals and—the fees stopped! She had no more attacks of colic. If she omits the use of soda for a length of time, say six or eight weeks, she will have a few premonitory twinges, the precursors of a more servere attack; but immediately obtaining a supply of the drug, she is safe so long as she continues to use it, and for some time afterward.

"This is not an isolated case, but one of several that could be adduced in favor of the use of phosphate of soda in the colic habit; but it is deemed useless to multiply instances upon so trivial a matter. Suffice it to say that I have never failed to see the administration of phosphate of soda followed by a complete cessation of the attacks of colic, and my experience in the use of this drug has now become quite extensive.

"In these cases I generally begin with thirty grains three times day, and if that amount produces much irritation of the bowels, indicated by frequent small discharges, attended perhaps by some tenesmus, I diminish the dose to twenty or fifteen grains. It is to be administered before meals, from a half to one hour, in a glass of water, and when thus dissolved it is not at all unpleasant to the taste.—*Drug Circular.*

**Suit for Damages.**—A curious suit against a physician (Boston Journal, April 2d, 1881), which illustrates one of the perils of the profession, was brought to a close lately in Philadelphia The facts were these: A man having been injured by a street-car passing over his limbs, a passing physician's carriage was stopped, and the physician—who happened to be Dr. William B. Atkinson, the affable Permanent Secretary of the American Medical Association—made to descend and give an opinion as to treatment. The advice given was to send for an ambulance and have the case taken to a hospital. The man dying a few days later, the widow brings suit against Dr. Atkinson for substantial damages for not yielding more prompt and efficient service to the patient, although no consideration had passed, and no special claim for such service shown to exist. When the case came up, Judge Ludlow ordered a non-suit to be entered for the plaintiff. Although this case did not go to a jury, where the issue would be uncertain, yet the defendant was put to considerable expense and loss of time, and a great deal of annoyance, by the prosecution, which made it an ordeal to which one would not be willingly subjected.

**Poisonous Ice.**—The Connecticut State Board of Health (Report for 1879) informs us that, in several instances, attention has been drawn to sewage-contaminated ponds with ice-houses upon their borders, and that several isolated cases of enteric disease, and one death, from the free use of ice polluted by sewage, have been recorded in that State during the year.

The curious natural experiment of the United States vessel, "Plymouth," an elaborate report of which was reviewed in the American Journal of the Medical Sciences for January, 1881, shows conclusively that the germs of yellow fever are not infallibly destroyed by a freezing, probably not by a zero temperature. Without venturing on any of the unsound reasoning from analogy, two common among medical theorists, this fact alone is sufficient to warn us of the possible danger that the poisons of enteric fever and other zymotic affections are not destroyed by the congelation of the water in which they float, even without the direct and positive testimony such as that given above that impure ice, especially when gathered from ponds polluted by sewage, may constitute a prolific cause of disease.—*News and Abstract.*

**Operative Interference in Gunshot Wounds of Peritoneum.**—* * * "In view of these facts, the writer ventures to advocate operative interference in gun-shot penetrating wounds of the peritoneum with any visceral leison, and similar cases without visceral injury. The wounds in the abdominal walls should be enlarged, or the linea alba opened freely enough to allow a thorough inspection of the injured parts. Hæmorrhage should be arrested. If intestinal wounds exist, they should be closed with animal ligatures, trimming their edges first if they are lacerated and ragged. Blood and all other extraneous matter should be carefully removed, and then provision made for drainage. If the wound of entrance is dependent, drainage may be secured by keeping this open. If the wound is a perforating one, and the apeture of exit dependent, the potency of this should be maintained, and, if necessary, a drainage tube of glass or other material introduced. If there is no wound of exit, and the wound of entrance is not dependent, then a dependent counter opening should be made and kept open with a drainage tube. If it is urged that the means suggested are desperate, it can be said in reply that the evil is desperate enough to justify the means.—*Maryland Med. Journal.*

**Psylium Seed in Constipation.**—We read in Paris Medical that Mr. Noel Gueneau de Mussy proposes using psylium or sarragota seed, besides white mustard seed, the use of which is excellent, or flax seed in the natural state.

Psylium is a species of plantain, commonly called fleawort, because of the appearance of its seeds, which are quite small, and very mucilaginous. A tablespoonful in half a glass of water is taken before dinner. He says that with a number of persons this method has proven as successful as with the Spanish lady from whom he obtained it. In other cases, however, he was obliged to alternate with more powerful laxatives, such as aloes or rhubarb, so as to keep up the effects. It is probable that psylium seed, like others of its kind, is not persistent in its effects, although in a number of cases it seems to have been so.—*Med. and Surg. Reporter.*

**Dangers of Chlorate of Potash.**—Speaking of diphtheria, Dr. Jacobi said: "In this connection I desire to say a final word in regard to large doses of chlorate of potassium, often recommended for diphtheria. My warnings in regard to this drug have at last been heeded. Extracts from my writings on this subject have been extensively published, and experiments on animals, made in Europe by Marchaud and others, have proved my clinical observation of the frequent occurrence of nephritis, and fatal nephritis, resulting from the incautious use of potassium chlorate. A number of fatal cases have been described, and it may be that much carelessness on the part of the public, and many accidents will be avoided in the future.—*Proceed. Amer. Med. Association.*

**French Treatment of Asthma.**—Dr. Evrard is said to have obtained very satisfactory results in a severe case of asthma from the use of sprays of iodide of potassium. The patient, a man thirty years of age, had suffered for eight months from daily attacks of asthma, and had also been subject to chronic bronchitis for five years. At the time the treatment began, he had three or four attacks a day, and was reduced to a pitiable condition. After assiduous use of the spray for eight days, the asthmatic attacks had almost entirely ceased. Eighteen months have elapsed since then, but the patient has continued to use the spray and the attacks have not returned. The strength of the solution used was 1 to 20.—*Ibid.*

**Mortality on the Globe.**—The Scalpel cites from the Gazette Medicale de Saint Petersburg, a curious calculation concerning the deaths and births of the earth. The population of the earth numbers 309 millions, of Asia 804 millions, of Africa 109 millions, and of America 85 millions. Taking as a basis for calculation the average mortality of France, which is comparatively low, because of its splendid hygienic and climatic conditions, the number of deaths for the whole globe is computed at 35,639,835 per year, 97,790 per day—67 per minute.

The number of births would be about 70 per minute, 100,800 per day, 36,792,000 per year.—*Lyon Medical.*

**A Triumph of Dentistry.**—At the last meeting of the Medical Society at Strasburg, reported in the Medical Gazette of Strasburg, Dr. Julius Bœckel presented, in the name of M. Sauval, dentist, a lady for whom the latter had extracted a small molar tooth for dental caries, with violent pain; and, having found it slightly carious to the bottom of its root, he sawed off the points of the root, filled it with gold carefully throughout the carious channel, and then reimplanted the tooth. The lady was free from all her pain; the tooth re-established itself solidly in the mouth; and, at the date at which she appeared at the Society (three weeks after the operation), the tooth served for mastication as well as her other teeth. This is certainly a remarkable example of what is technically described as dental autoprothesis with aurification.—*British Medical Journal*, Jan. 29, 1881.

**Cascara Sagrada, or Rhamnus Purshiana.**—I have used this drug for two years, and find it excellent for constipated bowels. When the rectum is torpid, and no other organ is affected, cold water enemata often restores regular passages, particularly after hard labors; but everything fails to act after one or two passages, and this drug proves a good remedy, and requires small doses. It is thought to act by toning the bowel to its natural function; but whatever the theory may be, its good effects have been acknowledged by hundreds of people wherever it has been used. When this remedy fails to act, as it does sometimes, I add one or two drops of the fluid extract of belladonna, and natural passages are obtained.—*Dr. Cullen in Med. Herald.*

**Whooping Cough.**—Dr. Mayes, in Journal of Materia Medica, says: I have found nothing else to relieve whooping cough so certainly and so quickly as quinine in small and frequent doses. In cases of young infants, 1-6 to 1-4 grain, repeated every two or three hours, will give decided relief in a few hours. For children of one year old, I prescribe 1-2 to 3-4 grain once in three hours. The cure is effected, or the disorder so much relieved as to be regarded as cured, in a week or ten days.

**Chloral Hydrate for Toothache.**—Dr. Sporea has found that chloral hydrate applied to a decayed tooth will often give instant relief. He puts three or four crystals (about 5 centigrammes) on a piece of cotton wool, and applies it direct.—*Eclectric Medical Journal.*

